# Autoantibody profile in sarcoidosis, analysis from the GRADS sarcoidosis cohort

**DOI:** 10.1371/journal.pone.0274381

**Published:** 2022-10-20

**Authors:** Basheer Khassawneh, Chengsong Zhu, Briana Barkes, Brian Vestal, Sarah Shrock, May Gillespie, Karin Pacheco, Kevin D. Deane, Lisa A. Maier, Quan-Zhen Li, Nabeel Hamzeh, GRADS investigators

**Affiliations:** 1 Jordan University of Science and Technology, Irbid, Jordan; 2 National Jewish Health, Denver, CO, United States of America; 3 University of Texas Southwestern Medical Center, Dallas, TX, United States of America; 4 University of Colorado, Aurora, CO, United States of America; 5 University of Iowa, Iowa City, IA, United States of America; University of Nebraska Medical Center, UNITED STATES

## Abstract

**Background:**

Sarcoidosis, a multi-systemic granulomatous disease, is a predominantly T-cell disease but evidence for a role for humoral immunity in disease pathogenesis is growing. Utilizing samples from the Genomic Research in Alpha-1 anti-trypsin Deficiency and Sarcoidosis (GRADS) study, we examined the prevalence of autoantibodies in sarcoidosis patients with pulmonary-only and extra-pulmonary organ involvement compared to normal controls.

**Study design and methods:**

We analyzed serum samples from sarcoidosis patients who participated in the GRADS study utilizing an autoantigen microarray platform for both IgM and IgG antibodies. The cohort included sarcoidosis patients with pulmonary-only disease (POS, n = 106), sarcoidosis patients with extra-pulmonary disease (EPS, n = 120) and a normal control cohort (NC, n = 101). Organ involvement was assessed following a standardized format across all GRADS participating centers.

**Results:**

Sarcoidosis patients overall had increased levels of IgM and IgG autoantibodies compared to normal controls. In addition, several autoantibodies were elevated in the POS and EPS cohorts compared to the NC cohort. Differences in autoantibody levels were also noted between the POS and the EPS cohorts. When comparing organ involvement with sarcoidosis, bone, spleen and ear, nose and throat involvement had higher IgM expression than other organs.

**Conclusion:**

Sarcoidosis patients have elevated IgM and IgG autoantibody levels compared to normal controls. In addition, individuals with pulmonary as well as additional organ involvement had higher IgM expression. Further research is needed focusing on specific organ-autoantibody pairs and role of autoantibodies in disease pathogenesis.

## Introduction

Sarcoidosis is a disease characterized by the formation of non-necrotizing granulomas that can involve any organ in the body in an unpredictable manner [1]. The current hypothesis is that sarcoidosis develops in a genetically predisposed individual who is exposed to yet unknown environmental trigger(s) [1]. The estimated prevalence of sarcoidosis in the US is at least 48/100,000 [2] and its mortality rate appears to be increasing [3].

There is an increased interest in the role of humoral immunity and autoimmunity in sarcoidosis [4–7]. Sarcoidosis patients are known to have hypergammaglobulinemia presumably due to non-specific stimulation of B-cells [8–12]. A high incidence of autoantibodies in the circulation has been reported in sarcoidosis patients with uveitis and autoimmune thyroiditis [13–15]. Autoimmune diseases such as systemic lupus erythematosus, systemic sclerosis, Sjogren’s syndrome, rheumatoid arthritis have been reported to co-occur in sarcoidosis patients [15–20]. By definition, autoantibodies target self-antigens which theoretically can explain extra-pulmonary organ involvement in sarcoidosis. The presence of autoantibodies and their association with extra-pulmonary organ involvement could serve as a potential diagnostic or risk assessment biomarker. It also points to the role of humoral immunity in the pathogenesis of sarcoidosis providing a novel target for therapy in sarcoidosis by targeting B-cells and antibody production.

Multiplex autoantigen arrays have been used to identify a wide spectrum of autoantibodies in patients with rheumatoid arthritis [21], systemic lupus erythematosus [22], autoimmune drug-induced liver injury [23] and chronic obstructive pulmonary disease [24]. Utilizing an autoantigen array platform, we investigated the profile of autoantibodies in the sera of sarcoidosis patients who were enrolled in the Genomic Research in Alpha-1 anti-trypsin Deficiency and Sarcoidosis (GRADS) study [25]. The GRADS sarcoidosis cohort is a well-defined group of sarcoidosis patients whose extra-pulmonary organ involvement was assessed utilizing a unified organ assessment tool across all participating centers. We examined the prevalence of autoantibodies in sarcoidosis patients with pulmonary-only, extra-pulmonary organ involvement compared to normal controls.

## Methodology

### Study population

We utilized the GRADS sarcoidosis cohort for this study [25]. GRADS was a multi-center study that recruited 368 sarcoidosis patients to investigate the association of lung microbiome and gene expression with disease phenotype [25]. All subjects underwent organ assessment based on their available clinical data utilizing a unified organ assessment tool. Serum samples were collected from all subjects at baseline and stored centrally in the Genomic Information Center of the study [25]. Cohorts were selected and divided based on organ involvement into those with pulmonary-only sarcoidosis (n = 106) or having evidence of extrapulmonary sarcoidosis (n = 120) with or without pulmonary disease based on the GRADS organ assessment tool. The number of subjects included was based primarily on the number of subjects in the pulmonary-only sarcoidosis cohort and funding limitations. All sarcoidosis patients in this cohort had no known autoimmune disease per the inclusion/exclusion criteria of the GRADS study [25]. Normal control samples (n = 101) were obtained from the National Jewish Health institutional biobank, a University of Colorado cohort, study of genetics, endotoxin and allergen (SAGE) cohort [26] and the Lung Tissue Research Consortium. All normal controls were not known to have any autoimmune disease at the time of sample collection. The study was approved by the National Jewish Health institutional review board (HS# 2814). All subjects consented to the original studies that collected the data and specimens but consent was waived by the IRB for this study as it was conducted on existing data and specimens.

### Autoantigen microarray platform

Autoantibody reactivities against a panel of autoantigens were measured using a pre-designed autoantigen microarray platform developed by University of Texas Southwestern Medical Center. We utilized an autoantigen microarray super panel which contains 128 autoantigens and internal controls. The autoantigens are listed on University of Texas Southwestern Medical Center Microarray and Immune Phenotyping core facility website (https://microarray.swmed.edu/products/product/autoantigen-microarray-super-panel-128-antigen-pan/). The serum samples were pretreated with DNAse-I to remove free DNA and then incubated with the autoantigen array at 1:50 dilution in PBS. Autoantibodies binding to the antigens were detected with cy3 labeled anti-human IgG and cy5 labeled anti-human IgM (Jackson Immunoresearch laboratory, USA, 1:1000 dilution) and Tiff images were generated using GenePix 4000B scanner with laser wavelengths 532nm for IgG and 635nm for IgM. Genepix Pro 6.0 software was used to analyze the images. The net fluorescence intensity (NFI) and signal-to-noise ratio (SNR) for each antigen were calculated by subtracting the background and negative control (PBS) effects and the averaged NFI and SNR were generated. SNR ≥ 3 were considered true signal from background noise and the antibodies with SNR < 3 in larger than 90% of all samples were filtered out. Antibody score (ABS) value was generated for each antibody using the following formular: ABS = log_2_[(NFI x SNR) + 1]

### Data analysis

Descriptive statistics of study cohorts were reported as means and SDs or percentages. Comparisons of categorical variables were completed using Fisher’s exact tests, whereas continuous variables were compared using Student t tests and Mann-Whitney U Test. Two-tailed nonparametric tests were used to compare ranks or differences between groups. Heatmaps were generated by Pheatmap R packages. R (3.5) was used for principal component analysis (PCA) with the Vegan (2.5) package. Adonis paired tests were performed to test the differences between groups. GraphPad prism (version 8.1) was also used for statistical tests. Leave-one-out cross-validation was performed, which involves using one observation as the validation set and the remaining samples as the training set. This is repeated until all samples are predicted by a stepwise multiple logistic regression model. Prediction performance was evaluated by the area under the curve (AUC) in the receiver operating characteristic (ROC) curve.

## Results

A total of 327 individuals, including 101 normal controls (NC), 106 pulmonary-only sarcoidosis (POS) and 120 extra-pulmonary sarcoidosis (EPS), were included in this analysis. A summary of the basic demographic characteristics for the cohorts is described in Table 1. For the extra-pulmonary sarcoidosis cohort, distribution of organ involvement is listed in Table 2. By definition, the pulmonary-only cohort had no clinical evidence of extra-pulmonary organ involvement.

**Table 1:** Demographics of the study cohorts.

	Pulmonary-only Sarcoidosis (n = 106)	Extra-Pulmonary Sarcoidosis (n = 120)	Normal Controls (n = 101)	p value
Age mean (SD)	51.7 (10.5)	53.8 (9.9)	51.6 (11.4)	0.33
Gender M/F percent	45/55	52/48	47/53	0.62
Race^&^				0.03
White	86(81%)	82(68%)	77(77%)	
Black	16(15%)	33(28%)	23(23%)	
Hispanic	0(0%)	3(2%)	0(0%)	
Asian	3(3%)	1(1%)	0(0%)	
IS Therapy percentage*	31%^#^	57%^$^	0%	<0.05

Abbreviations: SD: Standard deviation, M/F: Male/Female, IS: Immunosuppressive therapy.

*: The data for individual immunosuppressive dosing was not uniformly collected and could not be collated and reported.

&: Race was self-reported by subjects.

#: Two individuals had no data.

$: one individual had no data.

**Table 2:** Frequency of organ involvement in the extra-pulmonary sarcoidosis cohort.

Organ Involved	Percentage
**Pulmonary**	**95%**
**Cardiac**	**54%**
**Ophthalmic**	**46%**
**skin**	**25%**
**Joint**	**22%**
**Extra-thoracic lymphadenopathy**	**18%**
**Neurological**	**16%**
**ENT**	**14%**
**Calcium dysregulation**	**14%**
**Spleen**	**12%**
**Small fiber neuropathy**	**10%**
**Liver**	**9%**
**Salivary/lacrimal gland**	**9%**
**Muscle**	**7%**
**Bone**	**6%**
**Renal**	**3%**

Abbreviations: ENT: Ear, nose and throat

### Baseline autoantibody profile comparisons between the cohorts

**Fig 1:**
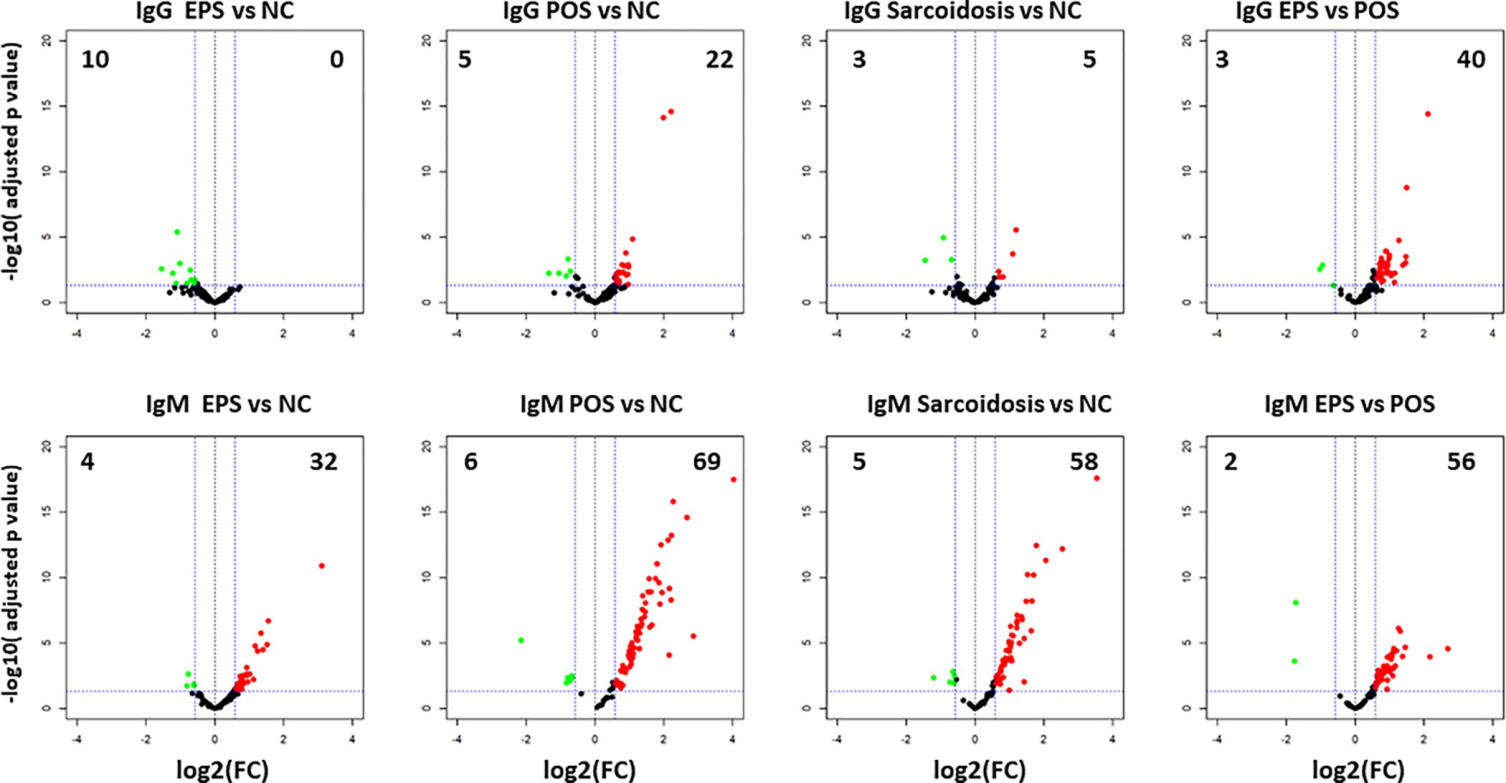
Autoantigen microarray analysis identified differential IgG and IgM autoantibody profiles in three groups (EPS: extra-pulmonary sarcoidosis; POS: pulmonary only sarcoidosis; NC: normal control; sarcoidosis is combination of EPS and POS). Sera from 120 EPS, 106 POS and 101 NC were assayed for autoantibodies using a high-throughput 120 autoantibody microarray platform. Volcano plots of IgG autoantibodies (top row) and IgM (bottom row) displaying each autoantibody as a single point with—log10(p value) on the y axis versus log2(FC) on the x axis. Red points indicate a statistically significant increase in mean autoantibody levels for each group comparison, green points indicate a statistically significant decrease for each group comparison. Fold changes are shortened as FC.

Out of the 120 autoantibodies assayed, 23 IgG autoantibodies and 35 IgM autoantibodies showed a low signal (SNR<3) in over 90% samples and were excluded from the analysis (S1 Table). There was no statistical difference in the antibody profile between groups based on age or sex. When we compared the entire sarcoidosis cohort (POS and EPS) to the NC cohort, only 5 IgG autoantibodies were significantly elevated in the sarcoidosis cohort compared to the NC cohort. Comparing each sarcoidosis cohort to the NC cohort, POS cohort had 22 autoantibodies significantly elevated whereas none were significantly elevated in the EPS cohort. Further comparison between the POS cohort and the EPS cohort identified 40 autoantibodies which were significantly elevated in the POS cohort vs. EPS cohort (Fig 1). This data indicating some IgG autoantibodies were elevated only in POS but not in EPS patients. Significantly different autoantibodies between cohorts are detailed in S2 Table.

Examining the IgM autoantibody profile revealed more significant differences. The combined sarcoidosis cohort had 58 significantly elevated IgM autoantibodies compared to the control group, while the POS and EPS alone showed 69 and 32 elevated IgM autoantibodies, respectively, comparing to the NC group (Fig 1), and among them, 31 are in common. Again, POS group exhibited more elevated IgM autoantibodies than EPS (Fig 1, S3 Table).

### Comparison of autoantibodies among the cohorts

**Fig 2:**
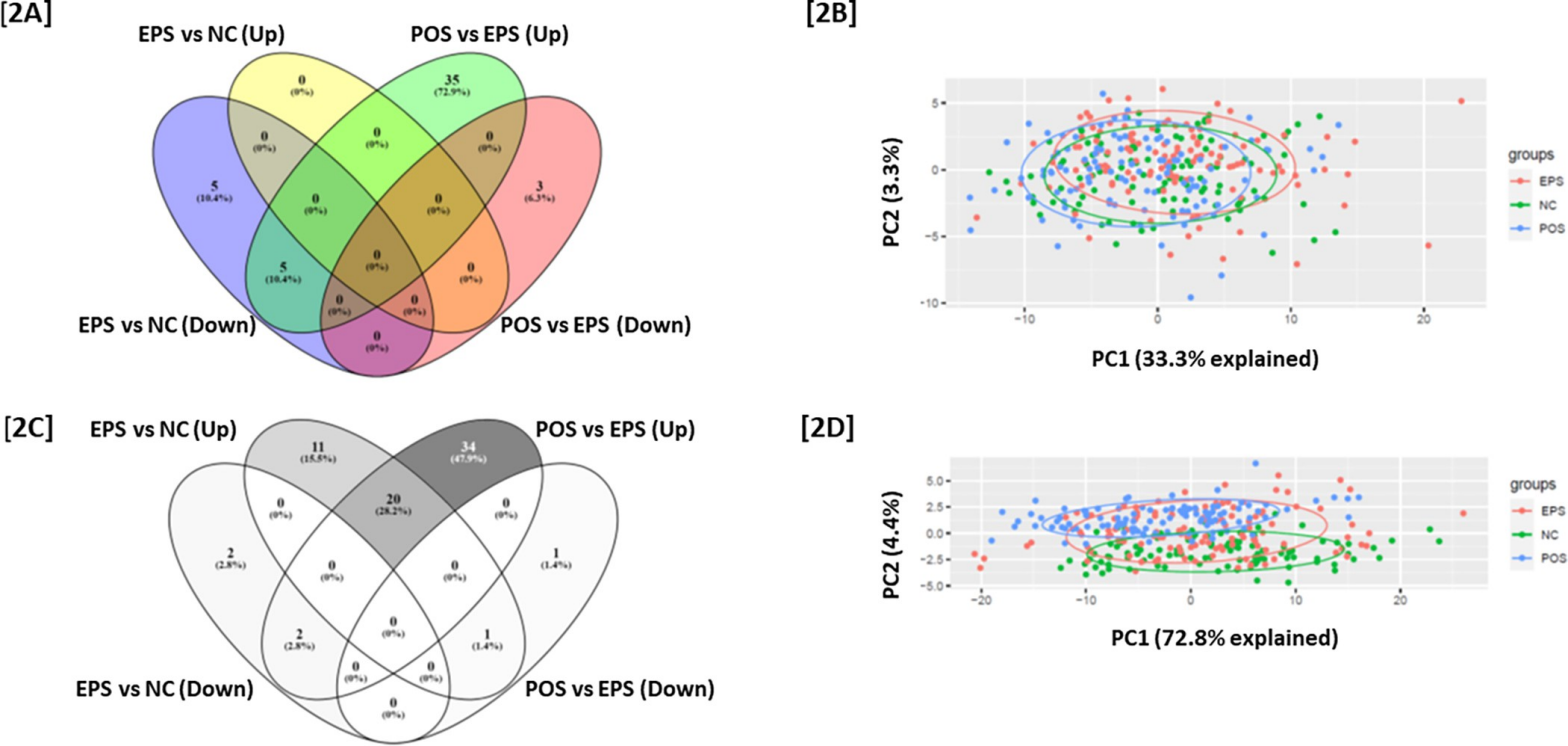
Patients with EPS showed significant increased IgM autoantibodies but not IgG autoantibodies compared with NC group (A) Venn diagram showing none is overlap between EPS versus NC and POS versus EPS (B) PCA of combined IgG autoantibodies showing clustering of POS difference from NC and EPS (pairwise Adonis test with adjusted p values for FDR: POS versus NC, p = 0.003; POS versus EPS, p = 0.003). EPS cluster is closer to NC cluster (adjusted p value by Adonis test is 0.035) (C) Venn diagram showing 20 elevated IgM antigens recognized all by EPS versus NCs and POS versus NC. (D) PCA of combined IgM autoantibodies showing clustering of POS difference from NC and EPS (pairwise Adonis test with adjusted p values for FDR: POS versus NC, p = 0.003; POS versus EPS, p = 0.003). EPS cluster is closer to NC cluster (adjusted p value by Adonis test is 0.027).

**Fig 3:**
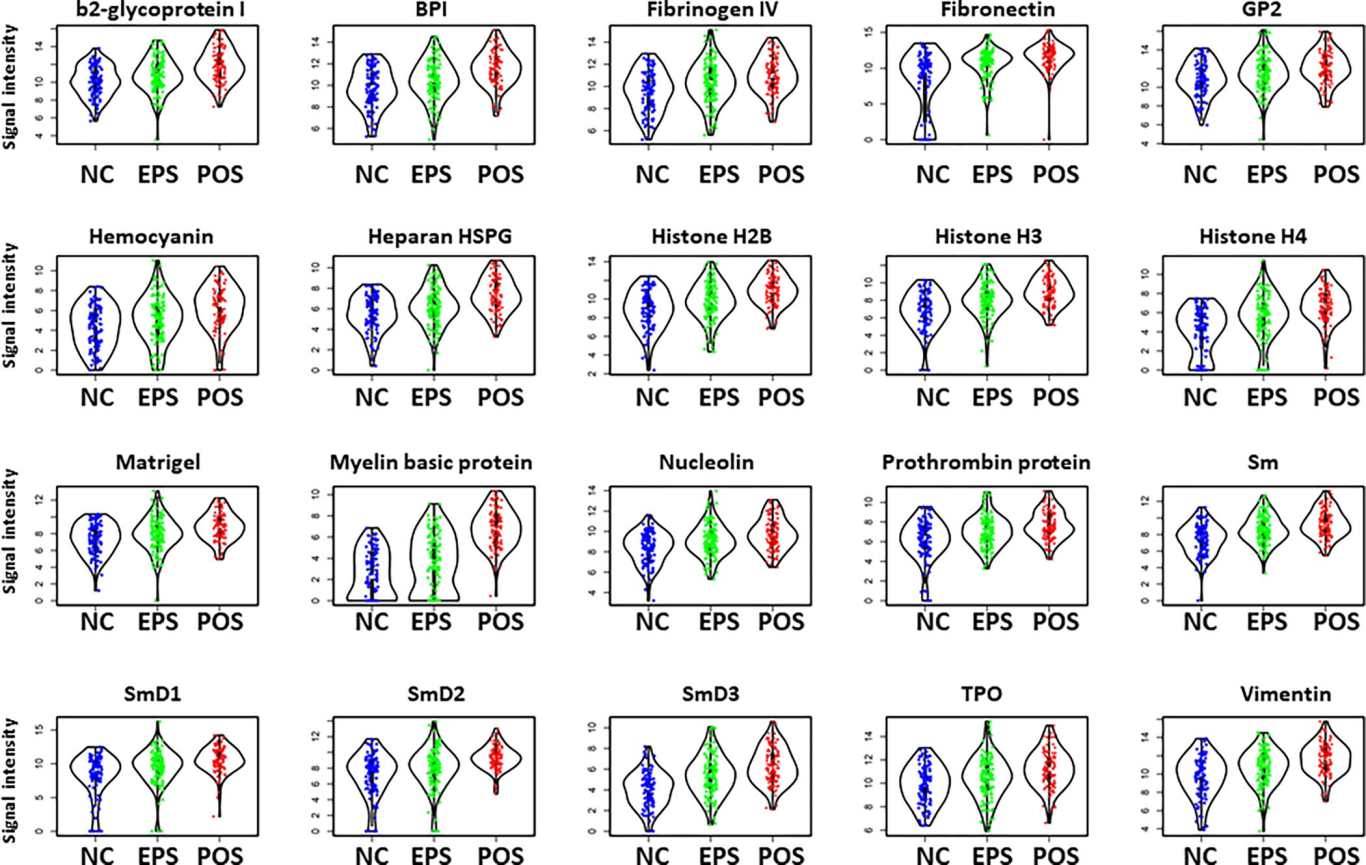
Violin plots generated by GraphPad prism 9 showed 20 IgM autoantibodies are significantly increased in EPS group compared to NC. Meanwhile, they are also significantly elevated in POS group compared with EPS group. Group comparisons were performed by Mann-Whitney U.

Principle component analysis (PCA) of IgG and IgM autoantibodies showed clustering of the POS cohort separated from the EPS and NC cohorts (Fig 2). The separation was more evident in the differential IgM autoantibodies compared to the IgG autoantibodies. Most of elevated IgM autoantibodies showed a trend of increase from EPS to POS. As shown in Fig 3, 20 IgM autoantibodies were identified to be significantly elevated in both EPS and POS, comparing with NC, but also with a significantly higher levels in POS than EPS.

### Autoantibodies for prediction of POS and EPS

**Fig 4:**
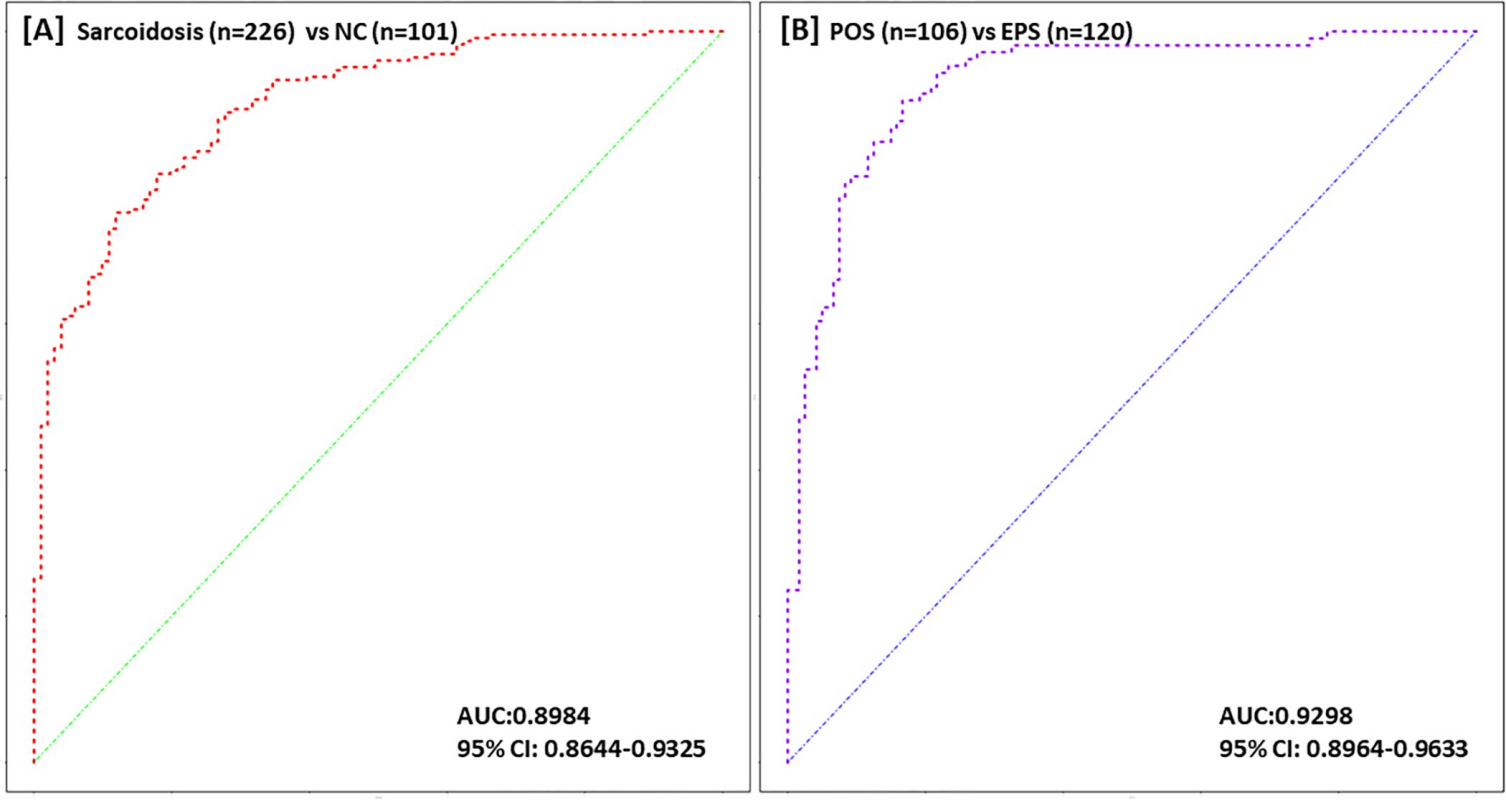
[A] ROC plots of 11 IgM autoantibodies are used as biomarkers to distinguish sarcoidosis (n = 226) from NC (n = 101) [B] ROC plots of 8 IgM autoantibodies can be applied to differentiate EPS from POS.

Stepwise multiple logistic regression analysis on the differentially expressed autoantibodies identified 11 IgM autoantibodies which can efficiently distinguish sarcoidosis patients from NC, with AUC 0.8984 and 95% CI: 0.8644~0.9325 (Fig 4A). Similar analysis also identified 8 IgM autoantibodies which showed best predictive value to distinguish POS from EPS, with AUC 0.9298, 95% CI: 0.8964~0.9633 (Fig 4B).

### Autoantibodies and specific organ involvement

The organ involvement in extra-pulmonary sarcoidosis cohort was determined based on the GRADS organ assessment tool that relied on clinically available records to define organ involvement(25). Sarcoidosis subjects with liver (p = 9.8×10^−4^), spleen (p = 4.5×10^−3^), bone and ear nose (p = 4.35×10^−5^) and throat (ENT) (p = 4.9×10^−3^) involvement had significantly higher IgM autoantibody values whereas those with skin (p = 1.3×10^−2^) and salivary/lacrimal gland (p = 4.4×10^−9^) involvement had significantly lower signal intensities (Table 3).

**Table 3:** Associations between IgM autoantibody profile and specific organ involvement.

IgM antibodies	Observed	HEART	EYE	SKIN	JOINT	LN	NEURO	ENT	CALCIUM	SPLEEN	LIVER	SFN	GLAND	MUSCLE	BONE	RENAL
Low	14 (11.67%)	9 (13.85%)	6 (10.91%)	6 (20%)	6 (23.08%)	6 (27.27%)	1 (4.76%)	1 (5.88%)	2 (11.76%)	2 (14.29%)	1 (7.69%)	0 (0.00%)	3 (27.27%)	0 (0.00%)	0 (0.00%)	1 (25%)
Slightly low	40 (33.33%)	23 (35.38%)	17 (30.91%)	12 (40%)	8 (30.77%)	7 (31.82%)	8 (38.10%)	4 (23.53%)	7 (41.18%)	3 (21.43%)	2 (15.38%)	5 (41.67%)	5 (45.46%)	4 (50.00%)	3 (42.86%)	0 (0%)
Moderate	32 (26.67%)	14 (21.54%)	14 (25.45%)	3 (10%)	6 (23.08%)	1 (4.55%)	7 (33.33%)	5 (29.41%)	3 (17.65%)	2 (14.29%)	3 (23.08%)	3 (25.00%)	0 (0.00%)	1 (12.50%)	1 (14.29%)	2 (50%)
High	34 (28.33%)	19 (29.23%)	18 (32.73%)	9 (30%)	6 (23.08%)	8 (36.36%)	5 (23.81%)	7 (41.18%)*	5 (29.41%)	7 (50.00%)*	7 (53.85%)*	4 (33.33%)	3 (27.27%)	3 (37.50%)	3 (42.86%)*	1 (25%)
Total	120	65	55	30	26	22	21	17	17	14	13	12	11	8	7	4

Abbreviations: LN: lymph node, ENT: Ear, nose and throat, SFN: small finer neuropathy.

*Liver: p = 9.8×10^−4^, spleen: p = 4.5×10^−3^, bone: p = 4.35×10^−5^ and ENT: p = 4.9×10^−3^ involvement had significantly higher IgM autoantibody values

## Discussion

There is increasing evidence that humoral immunity plays a role in the pathogenesis of sarcoidosis [5, 6, 13, 14, 27]. Our analysis of the autoantibody profile in sarcoidosis patients, recruited as part of the GRADS study, revealed the presence of increased autoantibody levels in both pulmonary-only and extra-pulmonary cohorts compared to healthy controls for both the IgM and IgG. In addition, the autoantibody profiles were different, with some overlap, between patients with pulmonary-only sarcoidosis and those with clinical evidence of extra-pulmonary sarcoidosis. In addition, when we examined the profiles in the context of organ involvement, patients with ENT, spleen and bone involvement, which are typically associated with chronic disease [28–30], had significantly higher IgM levels.

Evidence is slowly emerging to the potential role of B-cells in the pathogenesis of sarcoidosis. B-cell activating factor of the tumor necrosis family (BAFF) is produced by dendritic cells and monocytes/macrophages and is important for survival of immature B-cell and mature B-cell proliferation [27]. BAFF levels are elevated in sarcoidosis patients with active disease compared to inactive disease and the levels correlate with degree of hypergammaglobulinemia [27]. Our group recently demonstrated elevated levels of age-associated B-cells (ABCs) in the lavage and peripheral blood of sarcoidosis patients with higher levels noted in the BAL compared to the blood [5]. ABCs have been found to be elevated in a numer of infectious and autoimmune diseases [31]. Kamphius et al showed that B-cells and plasma cells are present on the outer shell of sarcoidosis granulomas and in a few cases within the granuloma [6]. These B-cells and plamsa cells are actively producing autoantibodies as Kinloch et al also demonstrated IgG and IgA anti-vimentin antibodies in the lung lavage fluid of sarcoidosis patients which was higher than that in matched serum samples suggesting local lung production of these antibodies which then overflow into the circulation [32]. In our cohort, IgM anti-vimentin antibodies was higher in both pulmonary-only and extra-pulmonary sarcoidosis compared to normal controls and higher in pulmonary-only compared to extra-pulmonary sarcoidosis cohort. IgG ant-vimentin was also higher in pulmonary-only sarcoidosis vs normal controls and extra-pulmonary sarcoidosis. All these data suggest a role for humoral immunity in sarcoidosis pathogenesis and suggest that autoantibodies may contribute to disease pthogenesis.

In our cohort, we detected both IgM and IgG autoantibodies against several antigens. Some of those targets overlapped and some were unique for each autoantibody class. IgM autoantibodies have been associated with several autoimmune diseases suggesting a potential role in disease manifestaiton [33–36]. What was surprising to us was that the sarcoidosis-pulmonary only cohort tended to have more positive antibodies than the extra-pulmonary cohort. A couple of possibilities could explain this finding; the presence of autoantibodies denotes risk of disease and not actual disease. In addition, the extent of organ involvement was based on standard of care evaluation of the patients at each center and was not based on a thorough systematic evaluation above and beyond standard of care, as such, occult subclinical organ invovlement could have been present in the pulmonary-only cohort.

Several of the autoantibodies noted in our study are pathogenic and have been associated with other systemic autoimmune diseases. For example, anti-Myelin Basic Protein (MBP) have been reported in multiple sclerosis and systemic lupus erythematosis (SLE) [37]. Anti-chromtin antibodies are also detected in SLE [38].Anti-histone antiboides target the protein components of nucleosomes and are common in SLE [39, 40], drug-indued lupus [41] and systemic sclerosis [42], Anti-LC1, liver cytosol type 1, antibodies are present in type 2 autoimmune liver disease [43], anti-glycoprotein 1 is an anti-phospholipid syndrome antibody which is associdated with increased risk of thrombosis [44], anti-SRP, signal recognition particle, has been reported in cases of necrotizing myopathy. It is plausable that autoantibodies that target self-antigens may bind to extra-pulmonary targets and initiate an immune response in that organ leading to granuloma formation. Our study is not large enough to detect the association of a specific antibody with a specific organ involvement in sarcoidosis but Tsukaka et al detected anti-endothelial cell antibodies in the sera of sarcoidosis patients and those antibodies were especially high in the sera of neurosarcoidosis patients [45]. Ten Berge et al investigated the presence of anti-retinal antibodies in the sera of patients with uveitis by indirect immunofluorescence. His cohort included 10 sarcoidosis patients with uveitis and 4 out of the 10 were postivie for anti-retinal antibodies [14]. We also recently showed evidence of anti-heart antibodies in sarcoidosis patients with cardiac sarcoidosis compared to sarcoidosis patients without evidence of cardiac sarcoidosis [46]. As such, organ specific autoantibodies may serve as potential biomarkers for risk stratification and guide intensity of organ surveillance.

The differential autoantibodies can be potential biomarkers for prediction of sarcoidosis and clinical subtypes. Using an regression algorithm, we identified a panel of 11 IgM autoantiboides including BPI, Fibrinogen IV, Hemocyanin, Histone H2B, Histone H3, Matrigel, Prothrombin protein, Sm, SmD, TPO and Vimentin, which can efficiently distinguish between sarcoidosis and healthy controls. Interestingly, anti-vimentin antibody has been previously proved to be associated with sarcoidosis [32]. A panel of 8 IgM autoantibodies were also been identified to have the best predictive value to distinguish between POS vs EPS.

Our data shows ovelap in the antibody profile between healthy controls and the extra-pulmonary sarcoidosis cohort. Extra-pulmonary sarcoidosis is very diverse in its presentation with variable organ manifestations. As the etiology of extra-pulmonary sarcoidosis is yet unknown (autoimmune phenomenon, antigen mimicry, direct antigen seeding of an organ (skin, eye)), it is plausible that some extra-pulmonary manifestations are autoimmune driven and some are not. As such, future studies focusing on specific-organ manifestations would be better powered to detect associations [46].

Our study has several limitations. First, although our autoantibody panel included over 100 antibodies, it is not an exhaustive panel. Second, the effect of immunosuppressive therapy may impact antibody levels although none of the patients in this cohort were on B-cell depleting therapies. Finally, the organ assessment was systematic and unified across the GRADS cohort but the assessment depended on clinically available tests and not on a thorough systemic organ assessment by advanced imaging or testing which leaves the door open to potential misclassification of organ involvement. All patients were seen and cared for at sarcoidosis specialty centers and underwent standard organ assessment evaluations and performing advanced studies, beyond standard of care, to fully investigate organ involvement is not feasible. Also, none of the sarcoidosis subjects had clinically known autoimmune disease as it was an exclusion criteria for the GRADS study.

In summary, humoral immunity is active in sarcoidosis and several autoantibodies are present in sarcoidosis patients and may play a role in the pathogenesis of pulmonary and extra-pulmonary sarcoidosis. Further research is needed to identify specific autoantibody-organ combinations if autoantibodies are to be used as a diagnostic and/or prognostic biomarker in sarcoidosis.

## Supporting information

S1 Table IgG and IgM autoantibodies excluded from analysis. (XLSX)

S2 Table IgG comparisons between sarcoidosis cohorts and normal controls. (XLSX)

S3 Table IgM comparisons between sarcoidosis cohorts and normal controls. (XLSX)

S1 Fig The heatmap of 120 IgG autoantigen shows reactivity expressed in terms of row z-score for a respective antigen across different patient samples. Each row in the graphics represent an antigen for serum specimens organized into columns classified as normal control (n = 101), extra pulmonary sarcoidosis (n = 120), and pulmonary only sarcoidosis (n = 106). The reactivity intensity ranges from blue (low) to white (moderate) or red (high).
(PPTX)

S2 Fig The heatmap of 120 IgM autoantigen shows reactivity expressed in terms of row z-score for a respective antigen across different patient samples. Each row in the graphics represent an antigen for serum specimens organized into columns classified as normal control (n = 101), extra pulmonary sarcoidosis (n = 120), and pulmonary only sarcoidosis (n = 106). The reactivity intensity ranges from blue (low) to white (moderate) or red (high).
(PPTX)

S3 Fig 120 extra pulmonary sarcoidosis subjects were classified into 4 subgroups based on hierarchical cluster analysis, the optimal number of clusters by a hierarchical cluster analysis was determined by k-means clustering. (PPTX)

## Abbreviation list

GRADS: Genomic Research in Alpha-1 anti-trypsin Deficiency and Sarcoidosis
NFI: Net fluorescence intensity
SNR: Signal to noise ratio
NC: Normal controls
SD: Standard deviation
BAFF: B-cell activating factor of the tumor necrosis family
EPS: Extra-pulmonary sarcoidosis
POS: Pulmonary-only sarcoidosis
NC: Normal control
ENT: Ear, nose and throat
ABC: Age-associated B-cells

## References

[1] Crouser ED, Maier LA, Wilson KC, Bonham CA, Morgenthau AS, Patterson KC, et al. Diagnosis and Detection of Sarcoidosis. An Official American Thoracic Society Clinical Practice Guideline. American journal of respiratory and critical care medicine. 2020;201(8):e26–e51. doi: 10.1164/rccm.202002-0251ST 32293205 PMC7159433

[2] Erdal BS, Clymer BD, Yildiz VO, Julian MW, Crouser ED. Unexpectedly high prevalence of sarcoidosis in a representative U.S. Metropolitan population. Respiratory medicine. 2012;106(6):893–9. doi: 10.1016/j.rmed.2012.02.007 22417737

[3] Swigris JJ, Olson AL, Huie TJ, Fernandez-Perez ER, Solomon J, Sprunger D, et al. Sarcoidosis-related mortality in the United States from 1988 to 2007. American journal of respiratory and critical care medicine. 2011;183(11):1524–30. doi: 10.1164/rccm.201010-1679OC 21330454 PMC3137141

[4] Ueda-Hayakawa I, Tanimura H, Osawa M, Iwasaka H, Ohe S, Yamazaki F, et al. Elevated serum BAFF levels in patients with sarcoidosis: association with disease activity. Rheumatology (Oxford, England). 2013;52(9):1658–66. doi: 10.1093/rheumatology/ket186 23685362

[5] Phalke S, Aviszus K, Rubtsova K, Rubtsov A, Barkes B, Powers L, et al. Age-associated B Cells Appear in Patients with Granulomatous Lung Diseases. American journal of respiratory and critical care medicine. 2020;202(7):1013–23. doi: 10.1164/rccm.201911-2151OC 32501729 PMC7528799

[6] Kamphuis LS, van Zelm MC, Lam KH, Rimmelzwaan GF, Baarsma GS, Dik WA, et al. Perigranuloma localization and abnormal maturation of B cells: emerging key players in sarcoidosis? Am J Respir Crit Care Med. 2013;187(4):406–16. doi: 10.1164/rccm.201206-1024OC 23239158

[7] Ando M, Goto A, Takeno Y, Yamasue M, Komiya K, Umeki K, et al. Significant elevation of the levels of B-cell activating factor (BAFF) in patients with sarcoidosis. Clin Rheumatol. 2018;37(10):2833–8. doi: 10.1007/s10067-018-4183-2 29936689

[8] Dall’Aglio PP, Pesci A, Bertorelli G, Brianti E, Scarpa S. Study of immune complexes in bronchoalveolar lavage fluids. Respiration; international review of thoracic diseases. 1988;54 Suppl 1:36–41. doi: 10.1159/000195495 3231904

[9] Daniele RP, McMillan LJ, Dauber JH, Rossman MD. Immune complexes in sarcoidosis: a correlation with activity and duration of disease. Chest. 1978;74(3):261–4. doi: 10.1378/chest.74.3.261 688781

[10] Fazel SB, Howie SE, Krajewski AS, Lamb D. B lymphocyte accumulations in human pulmonary sarcoidosis. Thorax. 1992;47(11):964–7. doi: 10.1136/thx.47.11.964 1465757 PMC464115

[11] Ghose T, Landrigan P, Asif A. Localization of immunoglobulin and complement in pulmonary sarcoid granulomas. Chest. 1974;66(3):264–8. doi: 10.1378/chest.66.3.264 4608492

[12] Gupta RC, Kueppers F, DeRemee RA, Huston KA, McDuffie FC. Pulmonary and extrapulmonary sarcoidosis in relation to circulating immune complexes: a quantification of immune complexes by two radioimmunoassays. The American review of respiratory disease. 1977;116(2):261–6. doi: 10.1164/arrd.1977.116.2.261 142432

[13] Amital H, Klemperer I, Blank M, Yassur Y, Palestine A, Nussenblatt RB, et al. Analysis of autoantibodies among patients with primary and secondary uveitis: high incidence in patients with sarcoidosis. International archives of allergy and immunology. 1992;99(1):34–6. doi: 10.1159/000236332 1483065

[14] Ten Berge JC, Schreurs MW, Vermeer J, Meester-Smoor MA, Rothova A. Prevalence and clinical impact of antiretinal antibodies in uveitis. Acta Ophthalmol. 2016;94(3):282–8. doi: 10.1111/aos.12939 26748893

[15] Nakamura H, Genma R, Mikami T, Kitahara A, Natsume H, Andoh S, et al. High incidence of positive autoantibodies against thyroid peroxidase and thyroglobulin in patients with sarcoidosis. Clinical endocrinology. 1997;46(4):467–72. doi: 10.1046/j.1365-2265.1997.1630976.x 9196610

[16] Begum S, Li C, Wedderburn LR, Blackwell V, Isenberg DA. Concurrence of sarcoidosis and systemic lupus erythematosus in three patients. Clin Exp Rheumatol. 2002;20(4):549–52. 12175113

[17] De Bandt M, Perrot S, Masson C, Meyer O. Systemic sclerosis and sarcoidosis, a report of five cases. Br J Rheumatol. 1997;36(1):117–9. doi: 10.1093/rheumatology/36.1.117 9117150

[18] Gal I, Kovacs J, Zeher M. Case series: coexistence of Sjogren’s syndrome and sarcoidosis. J Rheumatol. 2000;27(10):2507–10. 11036852

[19] Kucera RF. A possible association of rheumatoid arthritis and sarcoidosis. Chest. 1989;95(3):604–6. doi: 10.1378/chest.95.3.604 2920589

[20] Sharma OP. Sarcoidosis and other autoimmune disorders. Curr Opin Pulm Med. 2002;8(5):452–6. doi: 10.1097/00063198-200209000-00019 12172452

[21] Hueber W, Kidd BA, Tomooka BH, Lee BJ, Bruce B, Fries JF, et al. Antigen microarray profiling of autoantibodies in rheumatoid arthritis. Arthritis Rheum. 2005;52(9):2645–55. doi: 10.1002/art.21269 16142722

[22] Li QZ, Xie C, Wu T, Mackay M, Aranow C, Putterman C, et al. Identification of autoantibody clusters that best predict lupus disease activity using glomerular proteome arrays. J Clin Invest. 2005;115(12):3428–39. doi: 10.1172/JCI23587 16322790 PMC1297234

[23] Lammert C, Zhu C, Lian Y, Raman I, Eckert G, Li QZ, et al. Exploratory Study of Autoantibody Profiling in Drug-Induced Liver Injury with an Autoimmune Phenotype. Hepatol Commun. 2020;4(11):1651–63. doi: 10.1002/hep4.1582 33163835 PMC7603536

[24] Packard TA, Li QZ, Cosgrove GP, Bowler RP, Cambier JC. COPD is associated with production of autoantibodies to a broad spectrum of self-antigens, correlative with disease phenotype. Immunol Res. 2013;55(1–3):48–57. doi: 10.1007/s12026-012-8347-x 22941590 PMC3919062

[25] Moller DR, Koth LL, Maier LA, Morris A, Drake W, Rossman M, et al. Rationale and Design of the Genomic Research in Alpha-1 Antitrypsin Deficiency and Sarcoidosis (GRADS) Study. Sarcoidosis Protocol. Ann Am Thorac Soc. 2015;12(10):1561–71. doi: 10.1513/AnnalsATS.201503-172OT 26193069 PMC4627423

[26] Pacheco KA, McCammon C, Thorne PS, O’Neill ME, Liu AH, Martyny JW, et al. Characterization of endotoxin and mouse allergen exposures in mouse facilities and research laboratories. Ann Occup Hyg. 2006;50(6):563–72. doi: 10.1093/annhyg/mel019 16679338

[27] Saussine A, Tazi A, Feuillet S, Rybojad M, Juillard C, Bergeron A, et al. Active chronic sarcoidosis is characterized by increased transitional blood B cells, increased IL-10-producing regulatory B cells and high BAFF levels. PLoS One. 2012;7(8):e43588. doi: 10.1371/journal.pone.0043588 22927996 PMC3425471

[28] Wilcox A, Bharadwaj P, Sharma OP. Bone sarcoidosis. Curr Opin Rheumatol. 2000;12(4):321–30. doi: 10.1097/00002281-200007000-00016 10910186

[29] Zeitlin JF, Tami TA, Baughman R, Winget D. Nasal and sinus manifestations of sarcoidosis. Am J Rhinol. 2000;14(3):157–61. doi: 10.2500/105065800782102753 10887621

[30] Pavlovic-Popovic Z, Zaric B, Kosjerina Z, Petrovic D. Splenomegaly in Sarcoidosis: Frequency, Treatment, Prognosis and Long-term Follow-up. Srp Arh Celok Lek. 2015;143(5–6):279–83. doi: 10.2298/sarh1506279p 26259399

[31] Karnell JL, Kumar V, Wang J, Wang S, Voynova E, Ettinger R. Role of CD11c(+) T-bet(+) B cells in human health and disease. Cell Immunol. 2017;321:40–5. doi: 10.1016/j.cellimm.2017.05.008 28756897

[32] Kinloch AJ, Kaiser Y, Wolfgeher D, Ai J, Eklund A, Clark MR, et al. In Situ Humoral Immunity to Vimentin in HLA-DRB1*03(+) Patients With Pulmonary Sarcoidosis. Frontiers in Immunology. 2018;9(1516):1516. doi: 10.3389/fimmu.2018.01516 30038611 PMC6046378

[33] Arndt PA, Leger RM, Garratty G. Serologic findings in autoimmune hemolytic anemia associated with immunoglobulin M warm autoantibodies. Transfusion. 2009;49(2):235–42. doi: 10.1111/j.1537-2995.2008.01957.x 18980619

[34] Bernstein EJ, Barr RG, Austin JHM, Kawut SM, Raghu G, Sell JL, et al. Rheumatoid arthritis-associated autoantibodies and subclinical interstitial lung disease: the Multi-Ethnic Study of Atherosclerosis. Thorax. 2016;71(12):1082–90. doi: 10.1136/thoraxjnl-2016-208932 27609750 PMC5342945

[35] Filippidou N, Krashias G, Pericleous C, Rahman A, Ioannou Y, Giles I, et al. The association between IgG and IgM antibodies against cardiolipin, beta2-glycoprotein I and Domain I of beta2-glycoprotein I with disease profile in patients with multiple sclerosis. Mol Immunol. 2016;75:161–7. 27289032 10.1016/j.molimm.2016.05.022

[36] Laurent L, Anquetil F, Clavel C, Ndongo-Thiam N, Offer G, Miossec P, et al. IgM rheumatoid factor amplifies the inflammatory response of macrophages induced by the rheumatoid arthritis-specific immune complexes containing anticitrullinated protein antibodies. Ann Rheum Dis. 2015;74(7):1425–31. doi: 10.1136/annrheumdis-2013-204543 24618262

[37] Mario Gonzalez-Gronow SVP. Relevance of Catalytic Autoantibodies to Myelin Basic Protein (MBP) in Autoimmune Disorders. Journal of Neurology & Neuromedicine. 2018;3(4):4.

[38] Cervera R, Viñas O, Ramos-Casals M, Font J, García-Carrasco M, Sisó A, et al. Anti-chromatin antibodies in systemic lupus erythematosus: a useful marker for lupus nephropathy. Annals of the rheumatic diseases. 2003;62(5):431–4. doi: 10.1136/ard.62.5.431 12695155 PMC1754546

[39] Sui M, Lin Q, Xu Z, Han X, Xie R, Jia X, et al. Simultaneous positivity for anti-DNA, anti-nucleosome and anti-histone antibodies is a marker for more severe lupus nephritis. Journal of clinical immunology. 2013;33(2):378–87. doi: 10.1007/s10875-012-9825-6 23100145

[40] Shoenfeld Y, Segol O. Anti-histone antibodies in SLE and other autoimmune diseases. Clinical and experimental rheumatology. 1989;7(3):265–71. 2758706

[41] Vedove CD, Del Giglio M, Schena D, Girolomoni G. Drug-induced lupus erythematosus. Archives of dermatological research. 2009;301(1):99–105. doi: 10.1007/s00403-008-0895-5 18797892

[42] Hasegawa M, Sato S, Kikuchi K, Takehara K. Antigen specificity of antihistone antibodies in systemic sclerosis. Ann Rheum Dis. 1998;57(8):470–5. doi: 10.1136/ard.57.8.470 9797552 PMC1752726

[43] Liberal R, Grant CR, Longhi MS, Mieli-Vergani G, Vergani D. Diagnostic criteria of autoimmune hepatitis. Autoimmunity Reviews. 2014;13(4):435–40. doi: 10.1016/j.autrev.2013.11.009 24418295

[44] de Groot PG, Urbanus RT. The significance of autoantibodies against β2-glycoprotein I. Blood. 2012;120(2):266–74. doi: 10.1182/blood-2012-03-378646 22553312

[45] Tsukada N, Yanagisawa N, Mochizuki I. Endothelial cell damage in sarcoidosis and neurosarcoidosis: autoantibodies to endothelial cells. European neurology. 1995;35(2):108–12. doi: 10.1159/000117103 7796836

[46] Caforio ALP, Baritussio A, Marcolongo R, Cheng CY, Pontara E, Bison E, et al. Serum Anti-Heart and Anti-Intercalated Disk Autoantibodies: Novel Autoimmune Markers in Cardiac Sarcoidosis. J Clin Med. 2021;10(11):2476. doi: 10.3390/jcm10112476 34199661 PMC8199734

