# Supplementary material for: Autoantibody profile in sarcoidosis, analysis from the GRADS sarcoidosis cohort
Source: PLoS One. 2022 Oct 20;17(10):e0274381. doi: 10.1371/journal.pone.0274381 (PMC9584415; doi:10.1371/journal.pone.0274381)
Supplement: S2 Fig — Each row in the graphics represent an antigen for serum specimens organized into columns classified as normal control (n = 101), extra pulmonary sarcoidosis (n = 120), and pulmonary only sarcoidosis (n = 106). The reactivity intensity ranges from blue (low) to white (moderate) or red (high). (PPTX) [file pone.0274381.s005.pptx]

## Slide 1
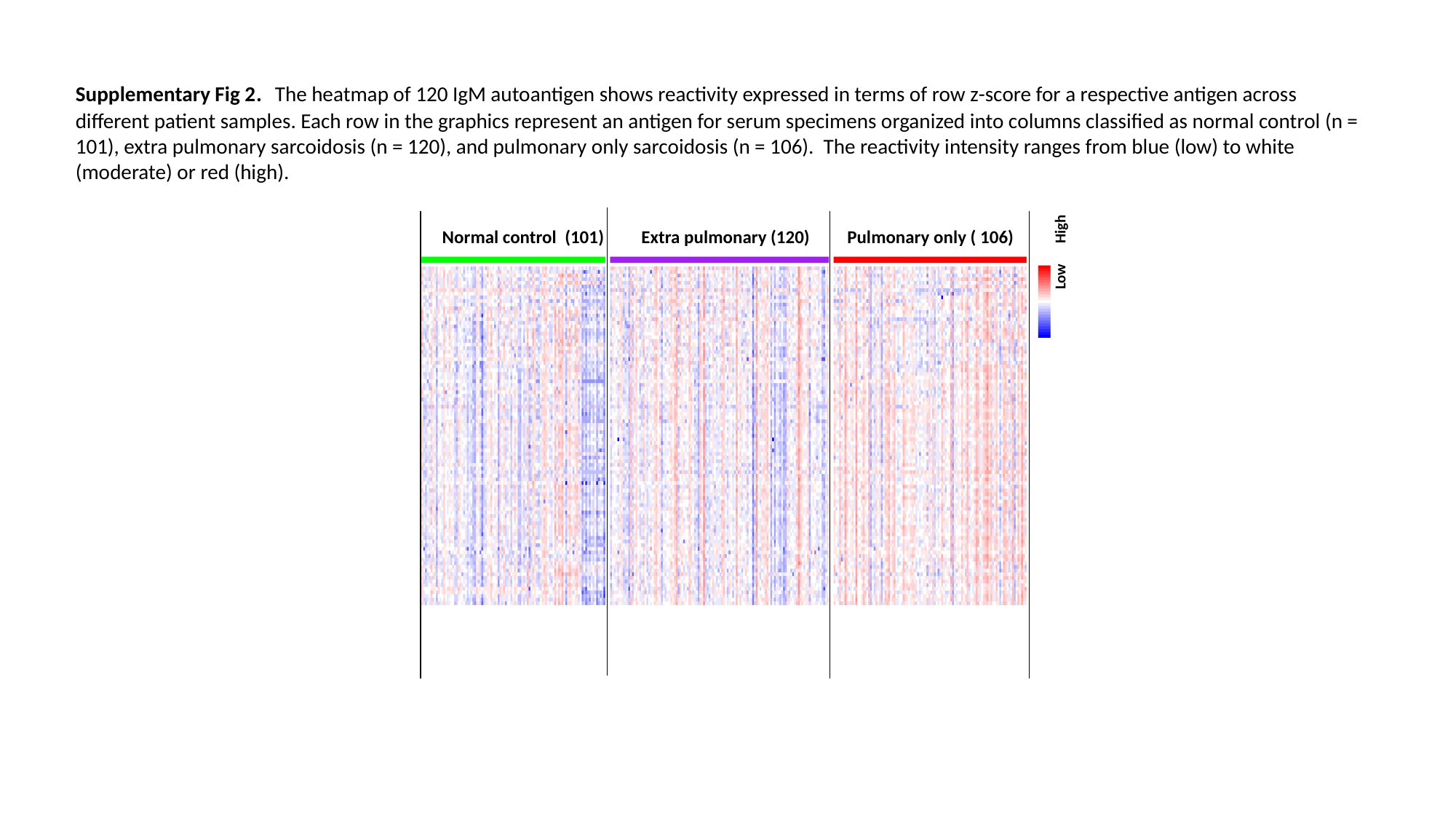

Supplementary Fig 2. The heatmap of 120 IgM autoantigen shows reactivity expressed in terms of row z-score for a respective antigen across different patient samples. Each row in the graphics represent an antigen for serum specimens organized into columns classified as normal control (n = 101), extra pulmonary sarcoidosis (n = 120), and pulmonary only sarcoidosis (n = 106). The reactivity intensity ranges from blue (low) to white (moderate) or red (high).
 Normal control (101) Extra pulmonary (120) Pulmonary only ( 106)
Low High
