# Supplementary material for: Autoantibody profile in sarcoidosis, analysis from the GRADS sarcoidosis cohort
Source: PLoS One. 2022 Oct 20;17(10):e0274381. doi: 10.1371/journal.pone.0274381 (PMC9584415; doi:10.1371/journal.pone.0274381)
Supplement: S3 Fig — (PPTX) [file pone.0274381.s006.pptx]

## Slide 1
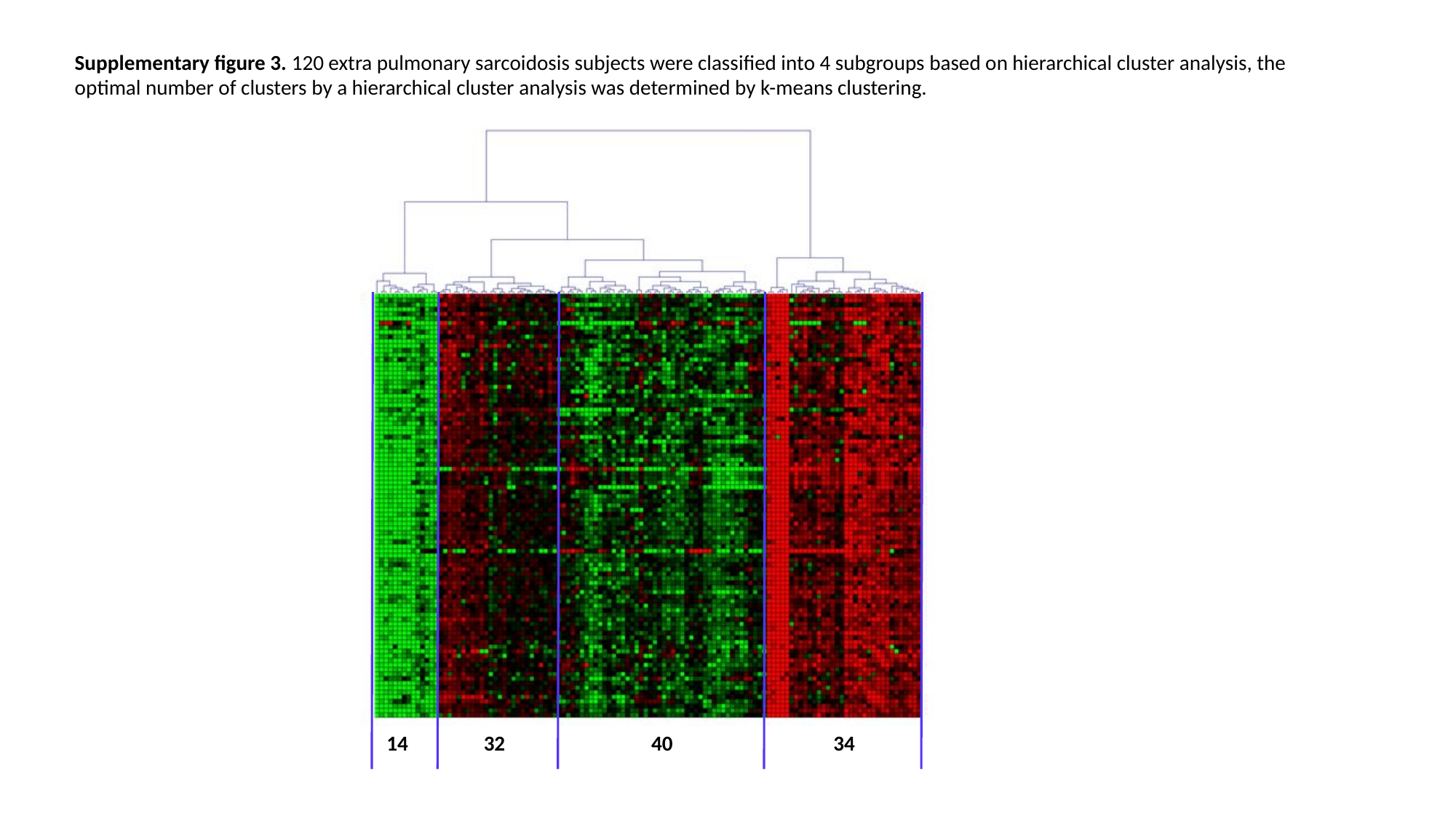

Supplementary figure 3. 120 extra pulmonary sarcoidosis subjects were classified into 4 subgroups based on hierarchical cluster analysis, the optimal number of clusters by a hierarchical cluster analysis was determined by k-means clustering.
 14 32 40 34
